# Sex-specific mortality differences in heart failure patients with ischemia receiving cardiac resynchronization therapy

**DOI:** 10.1371/journal.pone.0180513

**Published:** 2017-07-06

**Authors:** Zhonglin Han, Zheng Chen, Rongfang Lan, Wencheng Di, Xiaohong Li, Hongsong Yu, Wenqing Ji, Xinlin Zhang, Biao Xu, Wei Xu

**Affiliations:** Department of Cardiology, Nanjing University Medical School Affiliated Nanjing Drum Tower Hospital, Nanjing, China; University of Adelaide, AUSTRALIA

## Abstract

**Background:**

Recent studies have reported prognosis differences between male and female heart failure patients following cardiac resynchronization therapy (CRT). However, the potential clinical factors that underpin these differences remain to be elucidated.

**Methods:**

A meta-analysis was performed to investigate the factors that characterize sex-specific differences following CRT. This analysis involved searching the Medline (Pubmed source) and Embase databases in the period from January 1980 to September 2016.

**Results:**

Fifty-eight studies involving 33445 patients (23.08% of whom were women) were analyzed as part of this study. Only patients receiving CRT with follow-up greater than six months were included in our analysis. Compared with males, females exhibited a reduction of 33% (hazard ratio, 0.67; 95% confidence interval, 0.62–0.73; P < 0.0001) and 42% (hazard ratio, 0.58; 95% confidence interval, 0.46–0.74; P = 0.003) in all-cause mortality and heart failure hospitalization or heart failure, respectively. Following a stratified analysis of all-cause mortality, we observed that ischemic causes (p = 0.03) were likely to account for most of the sex-specific differences in relation to CRT.

**Conclusion:**

These data suggest that women have a reduced risk of all-cause mortality and heart failure hospitalization or heart failure following CRT. Based on the results from the stratified analysis, we observed more optimal outcomes for females with ischemic heart disease. Thus, ischemia are likely to play a role in sex-related differences associated with CRT in heart failure patients. Further studies are required to determine other indications and the potential mechanisms that might be associated with sex-specific CRT outcomes.

## Introduction

Cardiac resynchronization therapy (CRT) is an effective treatment for heart failure patients exhibiting wide QRS complexes and reduced systolic left ventricular ejection fractions (LVEF). Although CRT devices are routinely implanted according to ACCF/AHA/HRS guidelines, approximately 20% of CRT patients fail to benefit from CRT [1]. Recently, sex-specific differences in relation to heart failure (HF) epidemiology, clinical presentation, response to CRT, and post-CRT prognosis have been reported. However, the mechanisms that underlie these differences are not well understood. An AHA Statistical Update from 2016 [2] reported that heart failure mortality was higher in women than men; however, no obvious sex-specific differences were observed in relation to the prevalence of heart failure. Therefore, these data suggest that sex-specific factors may cause differences in CRT response between males and females. Indeed, most studies and meta-analyses performed in this area report that women who received CRT experienced greater benefits and reduced mortality compared with men [3]. Studies evaluating the differential effects of clinical factors, including ischemic events, left bundle branch block (LBBB), age, LVEF, and atrial fibrillation on male and female clinical outcomes are limited. Thus, it is important that we attempt to identify and characterize factors that might help us to improve clinical responses to CRT for both male and female HF patients.

The aim of this meta-analysis was to assess sex-specific differences in all-cause mortality in patients who received CRT. We also aimed to examine the effect of clinical factors on sex-related outcomes.

## Methods

This meta-analysis was performed according to the Preferred Reporting Items for Systematic Reviews and Meta-Analyses Statement [4]. No participation of human subjects were involved in this analysis.

### Literature search

A computer-based literature search by two reviewers (Z.H. and Z.C.) was performed to identify English-language publications listed in the Medline (Pubmed source) and Embase electronic databases from January 1980 to September 2016. The search terms and algorithm for the literature search were as follows: cardiac resynchronization therapy OR CRT OR pace maker AND (female OR women OR gender). The detailed search strategy were uploaded in supply materials (S1 File).

### Study selection

To further identify sex-specific differences in post-CRT prognosis, The selection of eligible studies was performed by two independent reviewers (Z.H. and Z.C.). The following studies or patients were included in this meta-analysis: (1) randomized controlled trials (RCT), prospective (PC) or retrospective (RC) studies, (2) studies that included patients with QRS ≥ 120 ms and LVEF ≤ 35%, (3) studies that compared endpoints for all-cause or any-cause mortality between males and females, (4) patients from studies with follow-up periods > 6 months. The causes of ischemia in associated cases included ischemic cardiomyopathy, ischemic heart failure, myocardial infraction, and patients who received coronary artery bypass grafting or stent implantation. Exclusion criteria for this analysis included the following: animal studies, case reports, review articles, meta-analyses, editorials, posters and studies that did not provide primary end-points for all-cause mortality or enough data to analyze sex-related differences. Disagreement was solved by discussion with another reviewer (W.X.).

### Quality assessment

The selection of eligible studies was performed by two independent reviewers (R.L. and W.D.). Since double-blinding is not possible in RCTs of pacemaker implantation, RCT study was evaluated using a modified version of the Jadad scale [5]. The quality of observational studies was assessed by Newcastle-Ottawa Quality Assessment Scale (NOS) [6], selection (a maximum of 4 points), comparability (a maximum of 2 points) and outcomes (a maximum of 3 points). To maintain quality control, only studies with more than 3 points (Jadad score) or 4 points (NOS score) were included in our analysis.

### Data extraction

Two reviewers (Z.H. and X.L.) independently extracted data from the associated studies. Results were compared and any disagreements were resolved by consensus. The following characteristics pertaining to the type of study design were extracted; author identification, published year, New York Heart Association functional class (NYHA), age, QRS duration, LVEF, morbidity of ischemic cause, atrial fibrillation and LBBB, and hazard ratio (HR) with 95% confidence interval (CI) for all-cause mortality and heart failure hospitalization or heart failure. In some studies, HR was not provided straightly, so the ratio was calculated by Tierney’s [7] method published in 2007. The primary endpoint of interest was death from any cause, and the secondary endpoint was heart failure hospitalization or heart failure.

### Publication bias and statistical analysis

Publication bias was assessed by author (X.Z. and W.J.) with funnel plot and by the using Stata 11.0. (Stata Corporation, College Station, TX) via Begg’s and Egger’s test of the intercept. Review Manage (RevMan) version 5.3 (Cochrane Collaboration, UK) was utilized for other statistical analyses by three authors (Z.H., Z.C. and H.Y.). HR and 95% CI values were calculated from the extracted data. All-cause mortality and heart failure hospitalization or heart failure were defined as the primary and secondary endpoints, respectively. Because of significant between-study heterogeneity, a random-effects model was employed to evaluate meta-regression models. Subgroups were categorized based on average values pertaining to age, female ratio, QRS duration, LVEF and average morbidity of ischemic events, atrial fibrillation and LBBB. The p-value of interaction was used to further investigate sex-differences among and between subgroups. P < 0.05 was considered to be statistically significant.

## Results

### Eligible trials

**Fig 1:**
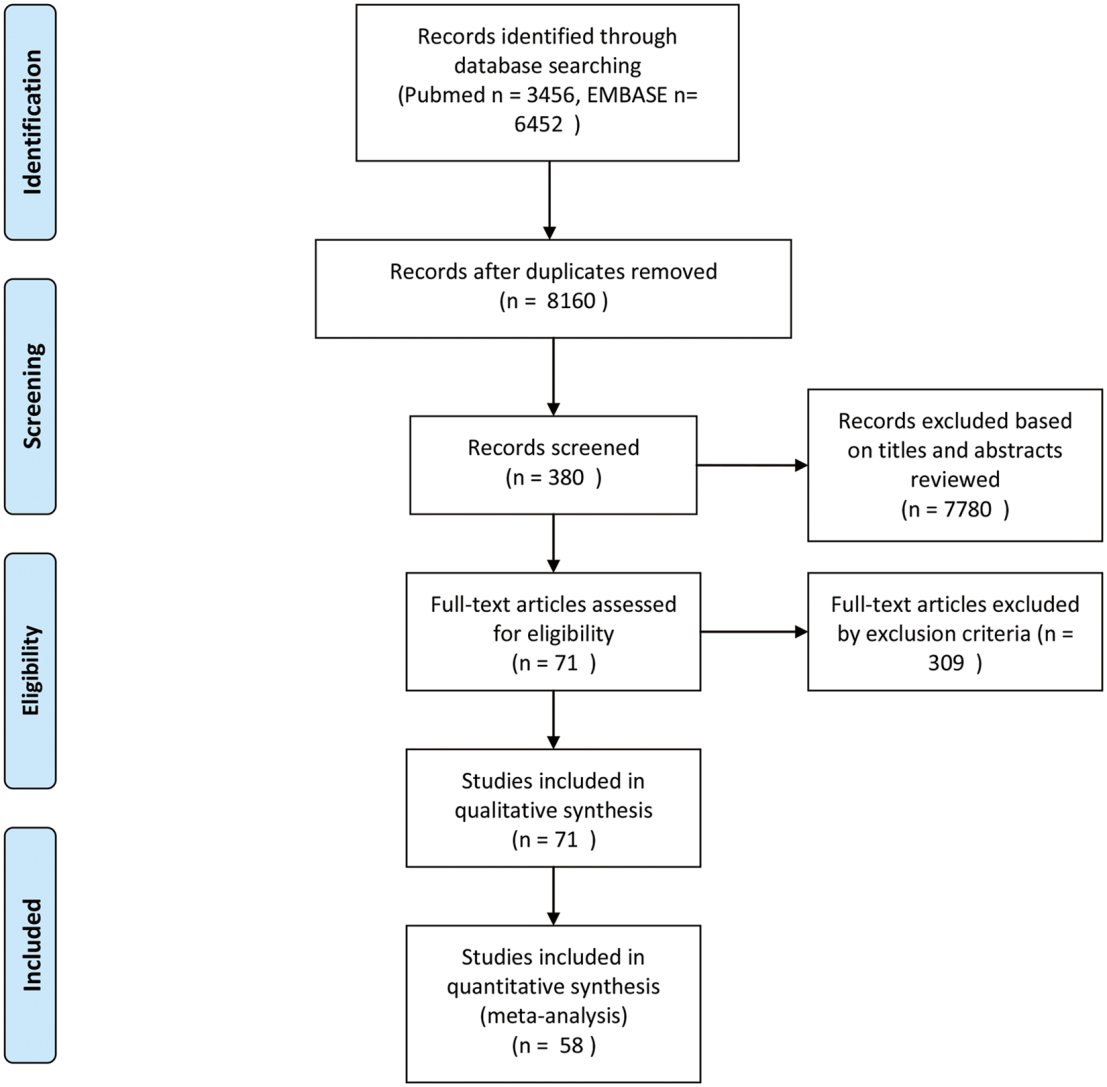
Flow chart of study design.

After duplicates removed, a total of 8160 citations were identified using the search strategy. Initially, after excluding non-relevant studies, reviews, meta-analyses, case reports, and a review of the abstracts, a total of 380 articles were identified. After quality assessment (studies with NOS score less than 4 points were excluded) and full text review, 58 studies were finally enrolled in our analysis as a result of full-text screening. The flow chart pertaining to the study selection is shown in Fig 1. All RCTs included in our analysis with Jadad score more than 3 points. And details of NOS scores distribution were shown in S1 Table.

### Baseline characteristics and outcomes

The fifty eight studies [8–65] that were included in this analysis reported sex-stratified relative risk estimates for all-cause mortality. A total of 34,455 individuals, 7952 (23.08%) of whom were females, were enrolled in these studies. All of the studies reported details regarding NYHA classification and 26 studies included NYHA class III-IV patients. A total of 23481 patients were categorized as NYHA class III-IV (78.40%) and 5532 individuals (18.46%) were classified as NYHA class I-II. Patients suffering from ischemia were identified in studies as those with ischemic cardiomyopathy, ischemic heart failure, myocardial infraction, and patients who received coronary artery bypass grafting or stent implantation. Mean values for age (67.19 years), QRS duration (161.46 ms) and LVEF (24.73%) were calculated from extracted data from 57, 52 and 54 studies, respectively. The average morbidity of patients with ischemic cardiomyopathy or related events, LBBB and AF was 51.39%, 72.17% and 25.48%, respectively; these data were extracted from 54, 40 and 22 studies, respectively. Additional characteristics and associated details including the type of study and follow-up durations are shown in Table 1. A total of 23.08% of the patients in the ischemic cause group were female. Similarly, 23.34% and 22.82% of the lower and higher risk subgroups were female, respectively. A total of 22.80% of the AF group was composed of female patients; 22.55% and 23.41% of the lower and higher risk subgroups were also comprised of female patients, respectively.

**Table 1:** Baseline characteristics for this meta-analysis.

Author (year)	Type	Total	Female	NYHA I/II	NYHA III/IV	Time	Age	ICs	LBBB	QRS (ms)	AF	LVEF (%)
Arshad (2011) [8]	RCT	1089	248	1089	-	12	64	-	-	-	210	-
Xu (2011) [9]	RC	728	166	-	723	44	69	316	333/712	165	213	24
Lilli (2007) [10]	RC	195	46	-	195	12	71	99	-	153	-	28
Mooyaart (2011) [11]	PC	578	147	-	578	24	67	342	406	166	99	23
Zardkoohi (2007) [12]	RC	117	26	-	117	24	71	80	-	169	-	20
Zabarovskaja (2012) [13]	RC	619	119	49	341	44	68	331	-	155	-	24
Cleland (2005) [14]	RCT	409	105	-	409	12	67	165	-	160	-	25
Schuchert (2013) [15]	RCT	393	82	4	389	24	68	194	-	164	44	25
Yu (2005) [16]	PC	141	38	12	129	6	-	-	-	-	-	-
Auricchio (2007) [17]	PC	1298	308	69	1208	34	64	561	-	168	243	24
Bai (2008) [18]	PC	542	124	-	542	27	66	361	-	162	268	20
Biase (2008) [19]	PC	398	99	-	398	53	67	219	-	187	-	22
Ei-saed (2009) [20]	RC	115	2	-	115	18	67	87	-	158	50	23
Fantoni (2008) [21]	RC	355	89	-	355	34	63	167	-	163	-	21
Iler (2008) [22]	RC	335	80	115/284	155/284	27	65	142/217	152/334	166	74/335	22
Kronborg (2008) [23]	RC	179	35	23	147	48	66	99	-	176	63	23
Shalaby (2008) [24]	RC	270	35	-	-	19	67	172	-	156	119	23
Stabile (2009) [25]	PC	233	53	26	207	58	69	114	-	-	51	27
Zhang (2009) [26]	PC	134	35	-	134	39	-	-	-	-	-	-
Delgado (2011) [27]	PC	397	56	-	397	21	67	397	-	155	-	25
Lin (2011) [28]	PC	482	98	-	-	37	71	-	-	164	-	29
Pfau (2010) [29]	RC	341	83	45	296	55	63	156	-	-	75	21
Foley (2011) [30]	RC	322	74	-	322	37	69	212	-	154	75	24
Miller (2011) [31]	RC	480	124	-	-	59	69	224	-	168	193	20
Prochnau (2011) [32]	PC	143	21	-	143	19	64	49	103	176	34	24
Rickard (2011) [33]	RC	668	193	-	-	50	66	387	-	159	348	22
Shen (2011) [34]	RC	100	27	-	100	17	70	66	-	165	-	20
Smit (2011) [35]	PC	338	88	32	306	27	65	170	-	164	145	24
Van (2011) [36]	PC	490	98	-	490	26	65	293	-	157	-	26
Eitel (2012) [37]	PC	219	40	-	219	56	67	150	119/151	180	61	23
Bogale (2012) [38]	PC	2111	488	415	1538	12	70	1006	1369	157	473	24
Kreuz (2012) [39]	RC	239	47	66	173	43	67	-	-	146	94	26
Morani (2013) [40]	PC	374	76	89	285	55	69	209	-	168	-	27
Risum (2013) [41]	RC	121	26	-	-	24	66	72	-	155	-	23
Rossi (2014) [42]	RC	330	66	99	231	55	62	135	-	161	53	28
Gasparini (2014) [43]	PC	3319	929	752	2567	37	67	1218	-	167	690	25
Reitan (2014) [44]	RC	446	76	58/398	340/398	82	72	262/435	280/443	170	268/446	25
Wilcox (2014) [45]	RCT	1023	318			24						
Sharma (2015) [46]	RC	511	116	124	387	22	69	294	-	161	146	24
Cipriani 1(2016) [47]	RC	507	101	186	321	48	62	198	406	161	73	27
Saxon (2006) [48]	RCT	595	196	-	595	16	66	327	434	159	-	23
Leyva (2011) [49]	pC	550	122	-	549	12	70	360	-	155	118	-
Looi (2014) [50]	RC	500	115	39	461	29	69	264	-	160	91	25
Perini (2014) [51]	RC	559	138	148	411	72	70	261	460	157	135	27
Khatib (2014) [52]	PC	600	122	135	465	36	67	253	-	168	155	25
Hoke (2014) [53]	RC	798	180	-	-	39	67	480	-	156	137	26
Lumens (2015) [54]	PC	191	50	-	-	24	66	115	115	159	-	24
Yanagisawa (2015)[55]	RC	125	34	-	125	37	67	35	70	161	14	26
Stabile (2015) [56]	PC	216	60	93	123	17	69	95	-	151	61	29
Roubicek (2015) [57]	PC	329	81	-	329	40	68	185	299	160	51	26
Rickard (2015) [58]	RC	723	210	-	723	60	67	439	-	165	384	21
Tayal (2015) [59]	PC	151	43	18	133	24	66	91	107	163	-	25
Nagy (2015) [60]	PC	93	21	12	81	24	67	46	84	160	26	30
Gold (2015) [61]	RCT	353	83	281	66	6	63	200	217	153	-	27
Gasparini (2015) [62]	PC	5153	1116	1159	3994	39	66	-	3441	158	899	26
Munir (2016) [63]	RC	512	149	-	-	41	81	264	236	154	-	23
Jacobsson (2016) [64]	RC	496	79	-	-	36	69	312	322	169	243	23
Cipriani 2(2016) [65]	PC	1122	247	316	806	12	66	527	774	153	356	28
Total	-	33445	7952	-	-	-	-	-	-	-	-	-
Average	-	-	-	18.46	78.40	-	67.19	51.39	72.17	161.47	25.20	24.73

ICs = ischemic causes.

**Fig 2:**
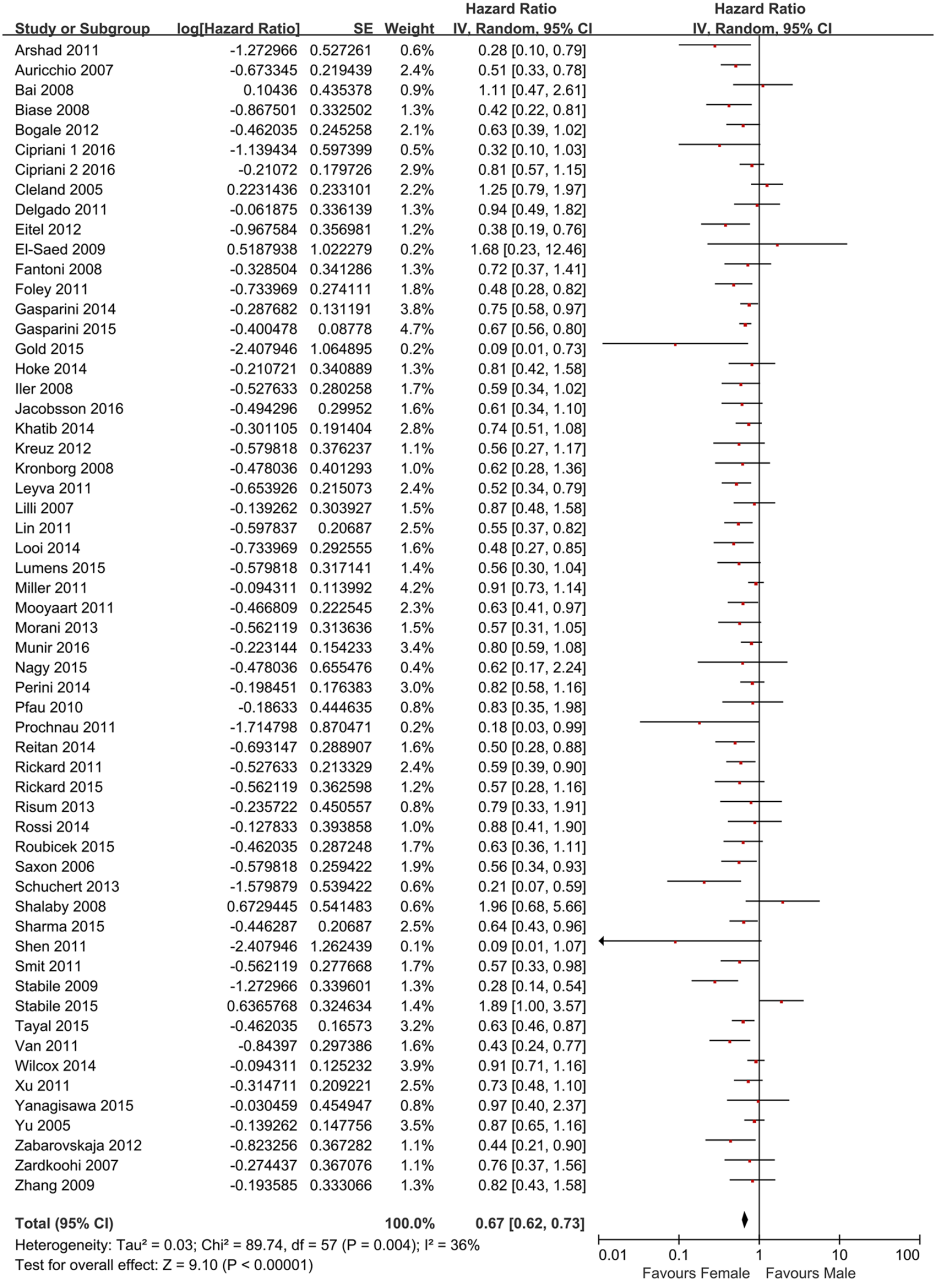
Forest plot for sex-specific differences in all-cause mortality of patients who received CRT.

**Fig 3:**
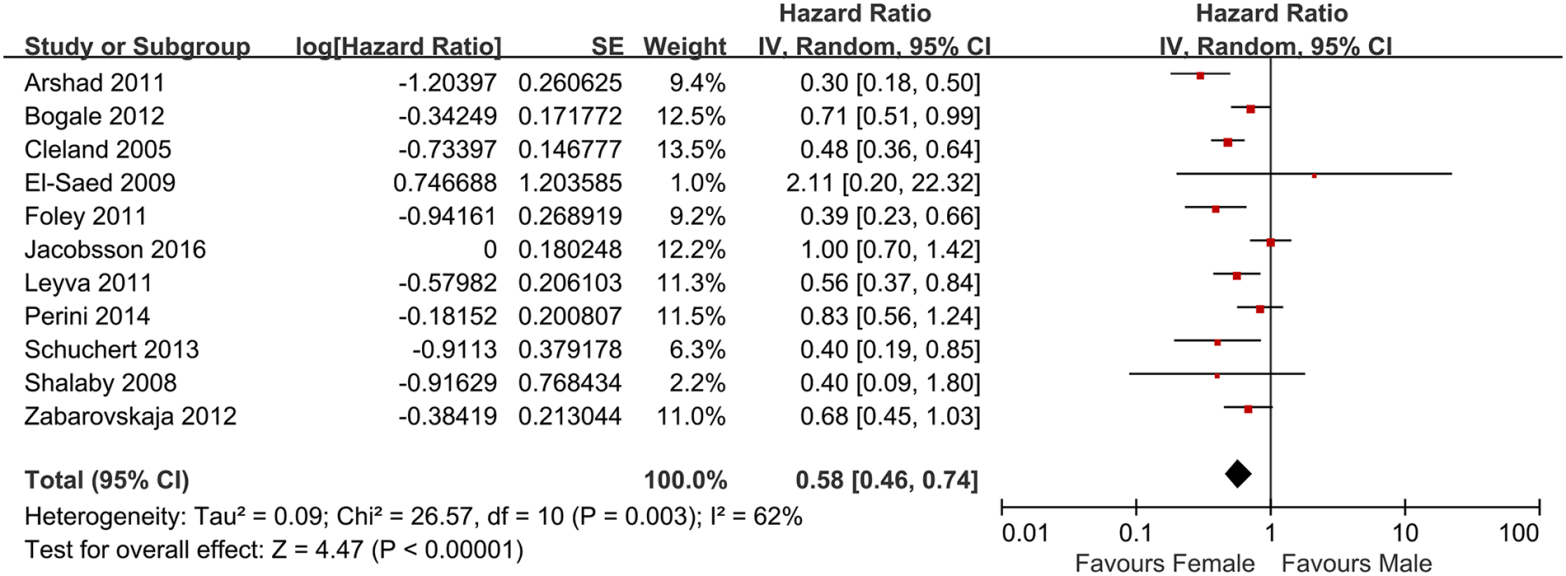
Forest plot for sex-specific differences in heart failure hospitalization or heart failure of patients who received CRT.

After pooling data from all of the studies, most of the studies (53/58) [8–14, 16, 17, 19, 21–23, 25–55, 57–65] reported that females exhibited better outcomes post-CRT compared with males. However, significant heterogeneity was observed among the 58 available studies (I^2^ = 36%; P = 0.004) and the HR for all causes mortality using random-effect models was 0.67 (95% CI, 0.62–0.73, P < 0.0001, Fig 2). Begg test showed no evidence of publication bias (P = 0.08) rather than Egger test (P = 0.01), the funnel plot was shown in S1A Fig. A total of 11 studies [8, 13–15, 20, 24, 30, 38, 49, 51, 64] reported sex-related differences in relation to heart failure hospitalization or heart failure; the HR for significant heterogeneity, which was calculated using random-effect models, was 0.58 (95% CI, 0.46–0.74, P < 0.0001, Fig 3) (I^2^ = 62%; P = 0.003). No evidence of publication bias was found in Begg test (P = 0.76) and Egger test (P = 0.68), and the funnel plot was shown in S1B Fig. To further investigate the reliable factors that led to significant sex-related differences for all-cause mortality, the NYHA class and average values pertaining to age, female ratio, QRS duration and LVEF were used to generate subgroups. In addition, the average morbidity associated with ischemic cause, LBBB and AF were used to establish lower and higher rate subgroups. The p-value of interaction between the ischemic cause subgroups was 0.03, no evidence of publication bias was found in Begg test (P = 0.81) and Egger test (P = 0.69) this may lead to the sex-related differences for all-cause mortality. Furthermore, the p-value of interaction between AF subgroups might also suggest an association with sex-related differences; however, no significant statistical difference was observed (P = 0.07). Furthermore, patients with ischemia or AF exhibited a 47% or 40% increase (HR) in risk in relation to all-cause mortality, respectively (associated details are shown in the S2 and S3 Figs, respectively), the funnel plots are shown in S4 Fig. Additional factors, including the type of study (P = 0.28), published year (P = 0.50), follow-up duration (P = 0.23), NYHA class (P = 0.77), age (P = 0.34), female ratio (P = 0.10), QRS duration (P = 0.28), LBBB (P = 0.66) and LVEF (P = 0.42), did not appear to statistically correlate with sex-related effects (Table 2).

**Table 2:** Stratified analysis of hazard ratio of all-cause mortality.

Subgroup		No. of studies	HR	IC		P value of interaction
Study type	RCT	6	0.55	0.33	0.94	0.28
	PC	25	0.65	0.57	0.73	
	RC	27	0.72	0.65	0.80	
Year	≤2010	16	0.72	0.58	0.88	0.50
	>2010	42	0.66	0.60	0.73	
Follow up	≤24 (m)	24	0.72	0.61	0.86	0.23
	>24 (m)	34	0.65	0.60	0.71	
Patients	≤500 (n)	20	0.69	0.63	0.76	0.58
	>500 (n)	38	0.66	0.58	0.76	
NYHA	I-V	32	0.66	0.60	0.74	0.77
	III-V	26	0.68	0.59	0.79	
Age	≤67.19 (y)	32	0.69	0.63	0.77	0.28
	>67.19 (y)	23	0.63	0.54	0.73	
Female	≤23.08(%)	30	0.62	0.56	0.69	0.10
	>23.08(%)	28	0.72	0.63	0.81	
Ischemic	≤51.39 (%)	22	0.74	0.64	0.86	0.03
	>51.39 (%)	31	0.61	0.55	0.67	
QRS	≤161.47 (ms)	29	0.72	0.64	0.81	0.28
	>161.47 (ms)	22	0.66	0.59	0.73	
LBBB	≤72.17 (%)	13	0.68	0.6	0.78	0.65
	>72.17 (%)	8	0.65	0.57	0.75	
AF	≤25.20 (%)	21	0.61	0.54	0.69	0.07
	>25.20 (%)	18	0.74	0.63	0.86	
LVEF	≤24.73 (%)	27	0.65	0.57	0.74	0.42
	>24.73 (%)	26	0.70	0.62	0.78	

The subgroups were based on average values pertaining to age, female ratio, QRS duration, LVEF and average morbidity of ischemic events, atrial fibrillation and LBBB. The average values for different characteristics associated with the subgroups are mentioned in Table 1.

## Discussion

The ACCF/AHA/HRS guidelines for CRT are based upon large clinical trials and meta-analyses of clinical trials. However, only 20% of the patients with HF that received CRT in these studies were female. This suggests that the current guidelines may be more applicable to male patients. In fact, clinical trial and meta-analysis data suggest that female patients respond better to CRT than male patients; however, there was no significant sex-related difference in relation to heart failure prevalence. More importantly, females exhibit higher mortality following HF, and were less likely to receive guideline-recommended treatment with medicine or CRT [66]. Data indicate that sex-related factors might influence the outcome of HF patients that receive CRT; however, this phenomenon has not yet been taken into account in the development of sex-specific diagnostic and treatment modalities, and we have yet to elucidate the potential clinical and molecular factors that underlie these sex-differences.

A total of 34,455 patients from 58 studies, of which 7952 (23.08%) were females, were included in our meta-analysis. For both primary and second endpoints, females exhibited a reduction of 33% and 42% in the rate of all-cause mortality and heart failure hospitalization or heart failure, respectively. These results are very similar to those presented in a previous meta-analysis by Cheng [67] in 2014. However, in the latter analysis, clinical factors including follow-up duration, proportions of female patients, NYHA function class, and average LVEF were not subject to sex-related differences. Indeed, only slight sex-related differences were found between European and American cohorts. Although geographical factors may have contributed to these observations, it is difficult to further analyze the sex-specific differences due to the lack of individual participant data.

Only a limited number of studies have analyzed sex-related differences or prognosis in patients with ischemic events, LBBB, age, LVEF or other clinical factors. It is possible that difference pertaining to the presentation of ischemic events is a factor that underpins sex-specific mortality. In our analysis, to further investigate potential factors that affect sex-specific outcomes, average values pertaining to age, female ratio, QRS duration and EF, along with mean morbidity values for ischemic cause, LBBB and AF were all used to establish subgroups. In summary, as a result of this meta-analysis it was observed that female patients suffering from ischemia experienced greater benefits following CRT in comparison with males with ischemia. Clinically, ischemic cardiomyopathy (ICM) is an independent risk factor for non-response in relation to CRT. However, patients with no ischemic cardiomyopathy (NICM) have been shown to exhibit better outcomes and greater reverse remodeling compared to those with ICM [68]. We further mined the collected data and analyzed the baselines associated with patients exhibiting ischemic causes and found: (1) ischemia is more common in males than in females (83.23% vs. 16.77%) [9–14, 19, 27, 38, 49], (2) the prevalence of female patients was similar in lower and higher rate ischemic cause subgroups (23.34% vs. 22.82%), (3) Patients with ischemia exhibited a 47% increase in the rate of all-cause mortality (S2 Fig). These findings suggest that male patients have a higher risk of ischemia, which is a certain factor in relation to higher mortality. Thus, the sex ratio of patients with ischemia is an important factor underlying differences pertaining to outcomes associated with CRT. Based on the higher morbidity rates in male patients, this result may explain why females benefit more and exhibit reduced mortality following CRT in comparison with males. Similarly, Herz et al [69] observed that sex-specific differences in CRT response may be the result of differences in the progression and presentation of heart failure; however, the exact nature of the baseline characteristics associated with this study was unclear.

Other clinical factors, including AF (P = 0.07), age, published year, QRS duration, NYHA class, and LVEF, did not correlate with sex-specific differences. Similar to ischemia, permanent AF was observed as attenuating the effect of CRT [70]. Additionally, in our analysis, AF resulted in a 40% increase in the risk of all-cause mortality during follow-up, and a higher prevalence of AF was observed in males compared with females (80.47% vs. 19.53%, respectively). However, we suggest that larger sample sizes should be analyzed to confirm this result. Importantly, female patients exhibit significantly higher complication rates during follow-up [71]. Females have higher blood pressure than males; they also exhibit a higher prevalence of heart failure with preserved ejection fraction. Both of these characteristics represent independent risk factors for stroke [72]. However, sex-specific complications are difficult to evaluate in clinical trials pertaining to CRT. Furthermore, the FDA have previously reported [73] that women are underrepresented in clinical trials due to fear of fetal consequences, associated economic costs, and lack of experience and knowledge in the management of disease during study follow-up.

Of note, without correcting for sex-specific differences in overall mortality on a population level, the benefit seen in women cannot be confidently associated with CRT. In most studies, survival was better for women with heart failure compared with men. And most patients enrolled in these studies were male. For example, in Chen’s meta analysis [67], 24.1% of subjects were women, but the causes for sex disparity were not evaluated. In our analysis, the HR of higher female ratio subgroup and lower female ratio subgroup were 0.72 and 0.62 respectively, and no significantly statistical difference (p = 0.10) was observed. However, it seemed that benefit of survival was attenuated with the increasing ratio of female patients. Thus, our results can not completely exclude the possibility that sex disparity play a potential role in sex-specific differences of survival. Further clinical trails are needed to answer the question whether women get more benefits after receiving CRT.

In addition to clinical factors, physiological factors are likely to influence the occurrence of sex-specific outcomes following CRT. As a consequence of smaller body mass and reduced ventricular size, female QRS durations are 5–10 ms shorter than for men [74]. Therefore, if this sex-specific difference in relation to QRS duration is taken into account, the net increase in conductive time of ventricles may be greater for females than males, especially in patients with complete LBBB. Furthermore, if the effect of body weight on QRS duration is normalized, a standardized QRS duration would help to improve patient selection for CRT, thereby providing a better prediction of CRT response[74]. Furthermore, females often exhibit greater age-related left ventricular concentric remodeling and higher EF compared with healthy aging men. Therefore, in addition to QRS duration and EF, which were both utilized as enrollment criteria, we speculate that greater conduction disturbance, ventricular dyssynchrony, and systolic dysfunction exist in female patients with HF who received CRT.

Additional cellular and molecular systems that are conventionally regulated by estrogen, including the autonomic nervous system [75], the extracellular matrix system [76] and the renin–angiotensin–aldosterone system (RAAS) [77], also participate in cardiac remodeling in female patients with HF. Although these factors could help us to understand how the response to CRT is affected by sex-specific effects, it would be difficult to implement associated factors into clinical guidelines [78]. However, the cellular and molecular mechanisms underpinning HF represent the theoretical basis of drug therapy for associated patients. Furthermore, rational drug therapy is extremely important for patients receiving CRT. Thus, it may be possible that there is a correlation between sex and CRT as a consequence of differences in post-CRT medicine responses. It is well known that all-cause mortality of HF patients receiving beta blockers and angiotensin converting enzyme inhibitors (ACEI) is significantly lower compared with those receiving placebo. Unfortunately, a meta-analysis [79] conducted by Kotecha reported no evidence of an interaction between beta blocker treatment effects and sex in any of the subgroups analyzed. Furthermore, Shekelle’s meta-analysis [80] revealed that women with asymptomatic LV systolic dysfunction may not have reduced mortality when treated with ACEI. However, a more recent study conducted by Kappert [81] reported a 20% lower risk for the combined cardiovascular end points in female patients with ACEI. Interestingly, similar to our finding, the latter sex-specific difference was driven primarily by a significantly lower incidence of myocardial infarction in female. This suggests that the nature of the ischemic cause plays a role in sex-related differences in relation to outcomes in HF patients. The mechanisms that underlie these differences warrant further studies to assess the relationship between molecular mechanisms and clinical effectiveness. However, in our analysis, it proved difficult to analyze sex-specific differences in relation to CRT effects without considering associated medicinal therapies.

Our meta analysis has some limitations. First, most HF studies stated that women have a survival advantage compared to men. Our analysis cannot exclude the possibility that the effects associated with ischemic patients may be due to generic survival capabilities as opposed to specific responses to CRT. Second, due to the fact that our study was limited with respect to quantitative data pertaining to sex-specific clinical factors, and the some studies included in our research with the NOS scores between 4 and 6, so the mean values and average morbidity data when categorizing the subgroups may not be so precise. Third, left ventricular lead location may influence the outcomes for HF patients receiving CRT; however, relatively little research has been conducted to investigate both location and sex-specific factors at the same time. At last, the publication bias was found in the analysis of all-cause mortality by Egger test, some small sizes studies enrolled in our analysis may be the potential reason. Thus, our results are limited and additional potential mechanisms that influence sex-related differences in CRT outcomes are still unclear.

In conclusion, similar to most clinical studies, the results from this meta-analysis suggest that female patients receiving CRT have a lower risk of all-cause mortality and heart failure or heart failure hospitalization. In addition, the occurrence of ischemia in patients might reduce the benefits associated with CRT. Furthermore, we observed that ischemia might cause differential sex-specific outcomes following CRT, especially in male patients with higher risks of ischemia. Further studies are required to elucidate the mechanisms that underpin sex-specific differences in relation to CRT.

## Supporting information

S1 File The details of search strategy. (DOCX)

S2 File PRISMA checklist for the present study. (DOC)

S1 Table NOS scores for the included non-RCT studies. (DOCX)

S1 Fig The funnel plots for all causes mortality and heart failure hospitalization or heart failure. (TIF)

S2 Fig Forest plot for all-cause mortality of ischemic causes for patients who received CRT. (TIF)

S3 Fig Forest plot for all-cause mortality of atrial fibrillation for patients who received CRT. (TIF)

S4 Fig The funnel plots for all-cause mortality of ischemic and atrial fibrillation. (TIF)
