# Supplementary material for: Sex-specific mortality differences in heart failure patients with ischemia receiving cardiac resynchronization therapy
Source: PLoS One. 2017 Jul 6;12(7):e0180513. doi: 10.1371/journal.pone.0180513 (PMC5500352; doi:10.1371/journal.pone.0180513)
Supplement: S1 File — (DOCX) [file pone.0180513.s001.docx]

Medline (Pubmed source): (cardiac resynchronization therapy OR pace maker AND (female OR women OR gender)) AND ("1980/01/01"[Date - Publication] : "2016/09/30"[Date - Publication]).

Embase electronic databases: cardiac AND resynchronization AND therapy AND (female OR women OR gender) AND [1-1-1980]/sd NOT [30-9-2016]/sd.
