# Supplementary material for: Sex-specific mortality differences in heart failure patients with ischemia receiving cardiac resynchronization therapy
Source: PLoS One. 2017 Jul 6;12(7):e0180513. doi: 10.1371/journal.pone.0180513 (PMC5500352; doi:10.1371/journal.pone.0180513)
Supplement: S1 Table — (DOCX) [file pone.0180513.s003.docx]

S1 Table. Modified Newcastle-Ottawa Scale (NOS) scores for the included non-RCT studies

|  | Selection | Comparability | Outcomes | Quality score |
| --- | --- | --- | --- | --- |
| Xu (2011) [8] | 3 | 2 | 1 | 6 |
| Lilli (2007) [9] | 3 | 0 | 1 | 4 |
| Mooyaart (2011) [10] | 4 | 2 | 2 | 8 |
| Zardkoohi (2007) [11] | 2 | 1 | 1 | 4 |
| Zabarovskaja (2012) [12] | 3 | 2 | 2 | 7 |
| Yu (2005) [15] | 4 | 0 | 2 | 6 |
| Auricchio (2007) [16] | 4 | 2 | 2 | 8 |
| Bai (2008) [17] | 3 | 1 | 0 | 4 |
| Biase (2008) [18] | 3 | 2 | 0 | 5 |
| Ei-saed (2009) [19] | 2 | 2 | 1 | 5 |
| Fantoni (2008) [20] | 2 | 2 | 2 | 6 |
| Iler (2008) [21] | 2 | 1 | 1 | 4 |
| Kronborg (2008) [22] | 2 | 2 | 2 | 6 |
| Shalaby (2008) [23] | 2 | 2 | 2 | 6 |
| Stabile (2009) [24] | 4 | 1 | 0 | 5 |
| Zhang (2009) [25] | 3 | 1 | 2 | 6 |
| Delgado (2011) [26] | 4 | 1 | 3 | 8 |
| Lin (2011) [27] | 4 | 2 | 1 | 7 |
| Pfau (2010) [28] | 2 | 1 | 2 | 5 |
| Foley (2011) [29] | 2 | 2 | 2 | 6 |
| Miller (2011) [30] | 2 | 2 | 2 | 6 |
| Prochnau (2011) [31] | 3 | 2 | 2 | 7 |
| Rickard (2011) [32] | 2 | 2 | 1 | 5 |
| Shen (2011) [33] | 2 | 1 | 1 | 4 |
| Smit (2011) [34] | 4 | 1 | 3 | 8 |
| Van (2011) [35] | 4 | 2 | 1 | 7 |
| Eitel (2012) [36] | 3 | 2 | 2 | 7 |
| Bogale (2012) [37] | 3 | 1 | 2 | 6 |
| Kreuz (2012) [38] | 2 | 1 | 1 | 4 |
| Morani (2013) [39] | 3 | 2 | 1 | 6 |
| Risum (2013) [40] | 2 | 1 | 2 | 5 |
| Rossi (2014) [41] | 2 | 1 | 1 | 4 |
| Gasparini (2014) [42] | 3 | 2 | 0 | 5 |
| Reitan (2014) [43] | 2 | 2 | 1 | 5 |
| Sharma (2015) [45] | 2 | 2 | 1 | 5 |
| Cipriani 1(2016) [46] | 2 | 2 | 1 | 5 |
| Leyva (2011) [48] | 3 | 1 | 1 | 5 |
| Looi (2014) [49] | 2 | 2 | 1 | 5 |
| Perini (2014) [50] | 2 | 1 | 1 | 4 |
| Khatib (2014) [51] | 4 | 1 | 1 | 6 |
| Hoke (2014) [52] | 2 | 2 | 1 | 5 |
| Lumens (2015) [53] | 4 | 1 | 3 | 8 |
| Yanagisawa (2015) [54] | 2 | 2 | 1 | 5 |
| Stabile (2015) [55] | 3 | 1 | 1 | 5 |
| Roubicek (2015) [56] | 4 | 0 | 2 | 6 |
| Rickard (2015) [57] | 2 | 2 | 1 | 5 |
| Tayal (2015) [58] | 4 | 2 | 3 | 9 |
| Nagy (2015) [59] | 4 | 1 | 0 | 5 |
| Gasparini (2015) [61] | 4 | 2 | 0 | 6 |
| Munir (2016) [62] | 2 | 2 | 1 | 5 |
| Jacobsson (2016) [63] | 2 | 1 | 2 | 5 |
| Cipriani 2(2016) [64] | 4 | 1 | 2 | 7 |
