# Supplementary figures and images for: Sex-specific mortality differences in heart failure patients with ischemia receiving cardiac resynchronization therapy
Source: PLoS One. 2017 Jul 6;12(7):e0180513. doi: 10.1371/journal.pone.0180513 (PMC5500352; doi:10.1371/journal.pone.0180513)

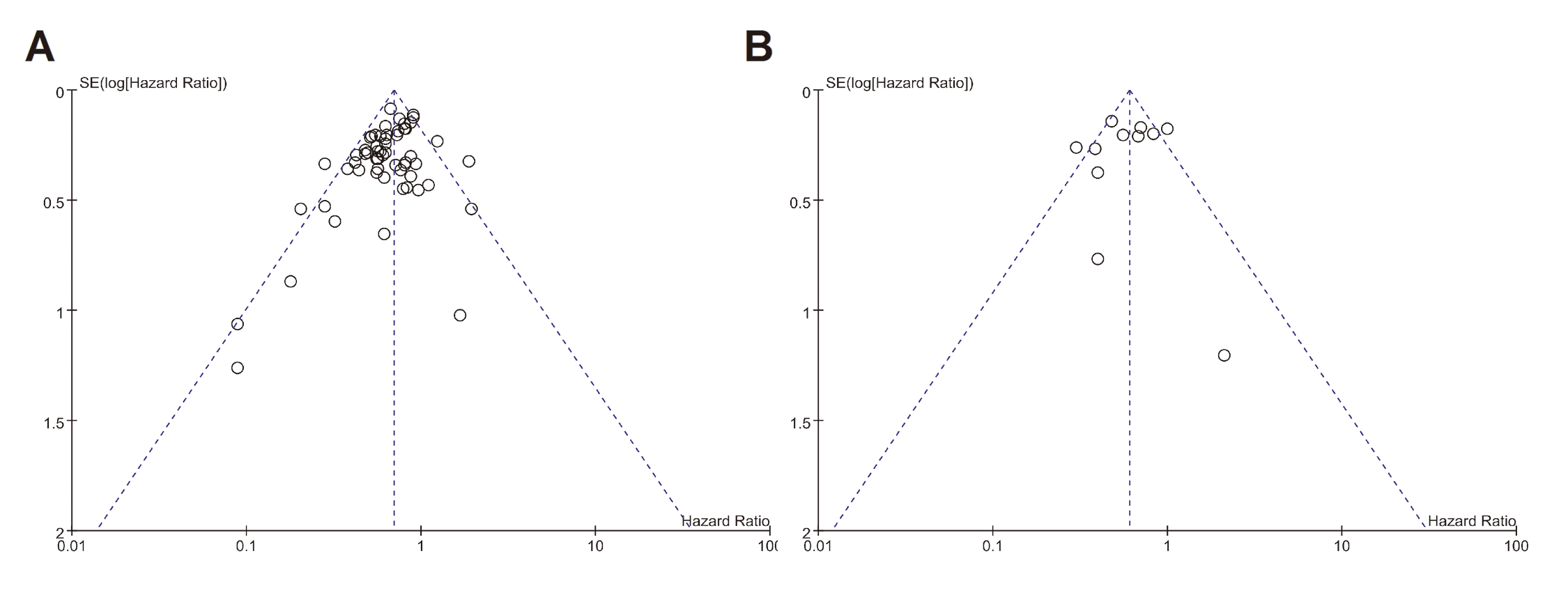

Supplement: S1 Fig — (TIF) [file pone.0180513.s004.tif]

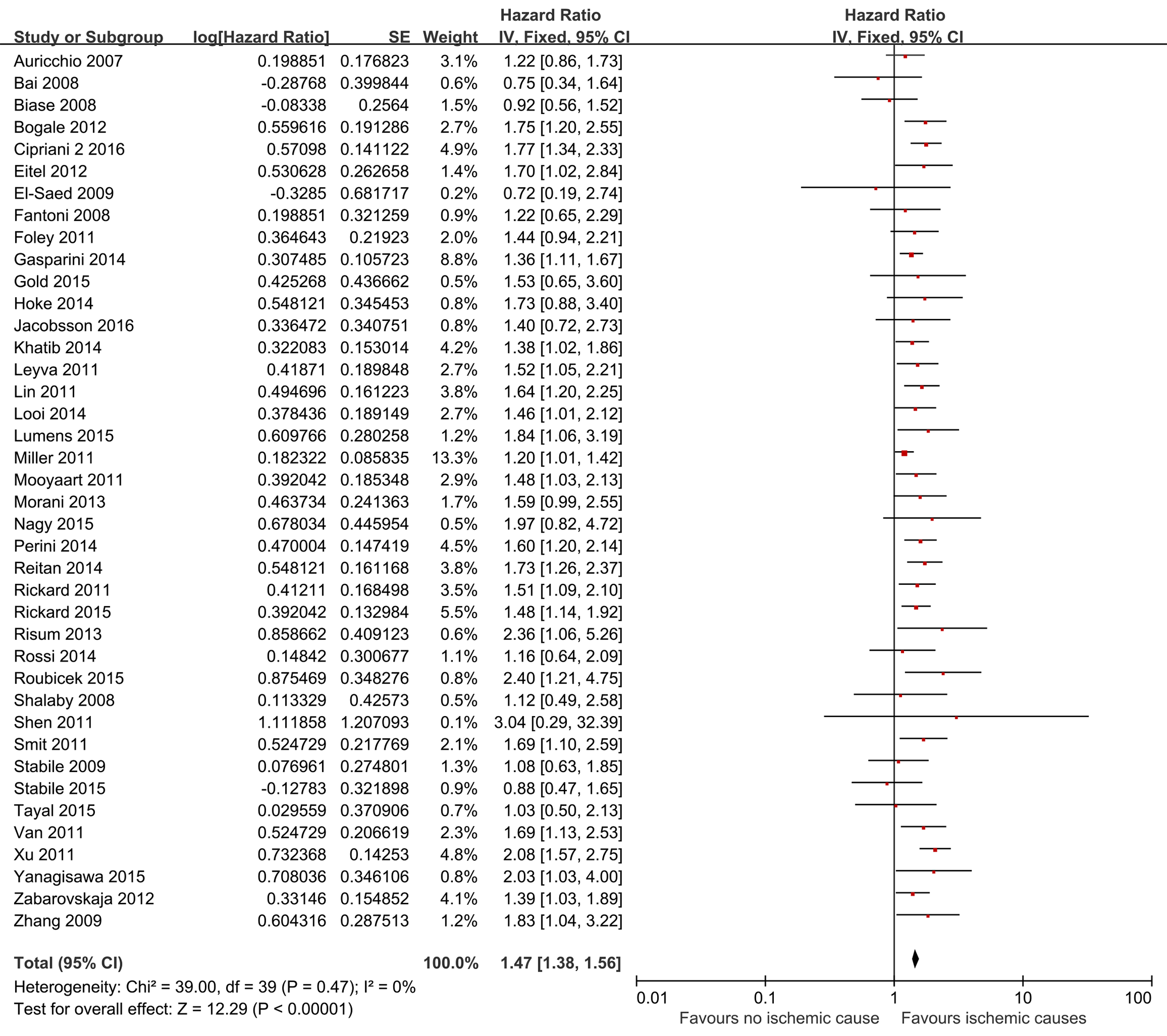

Supplement: S2 Fig — (TIF) [file pone.0180513.s005.tif]

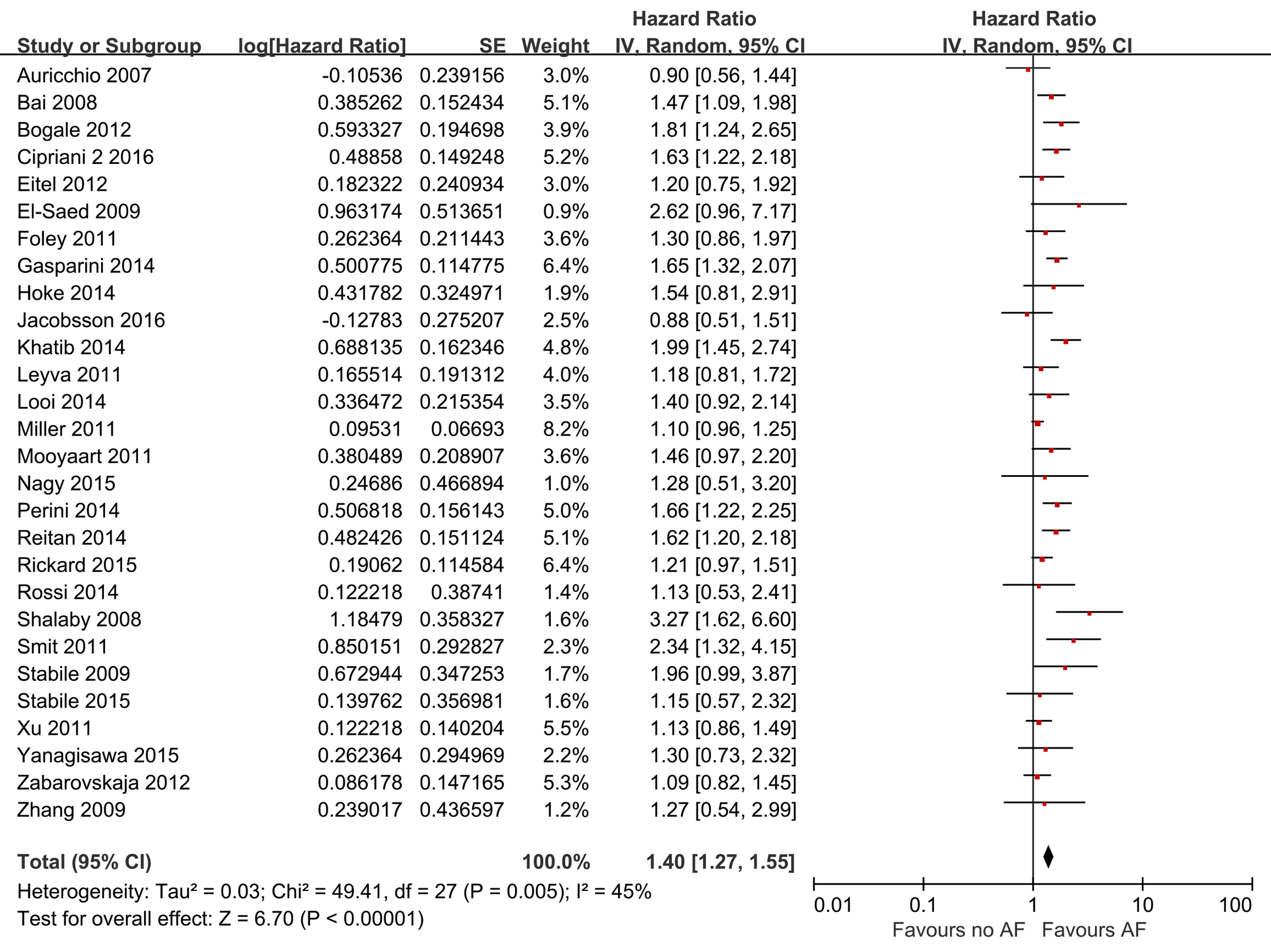

Supplement: S3 Fig — (TIF) [file pone.0180513.s006.tif]

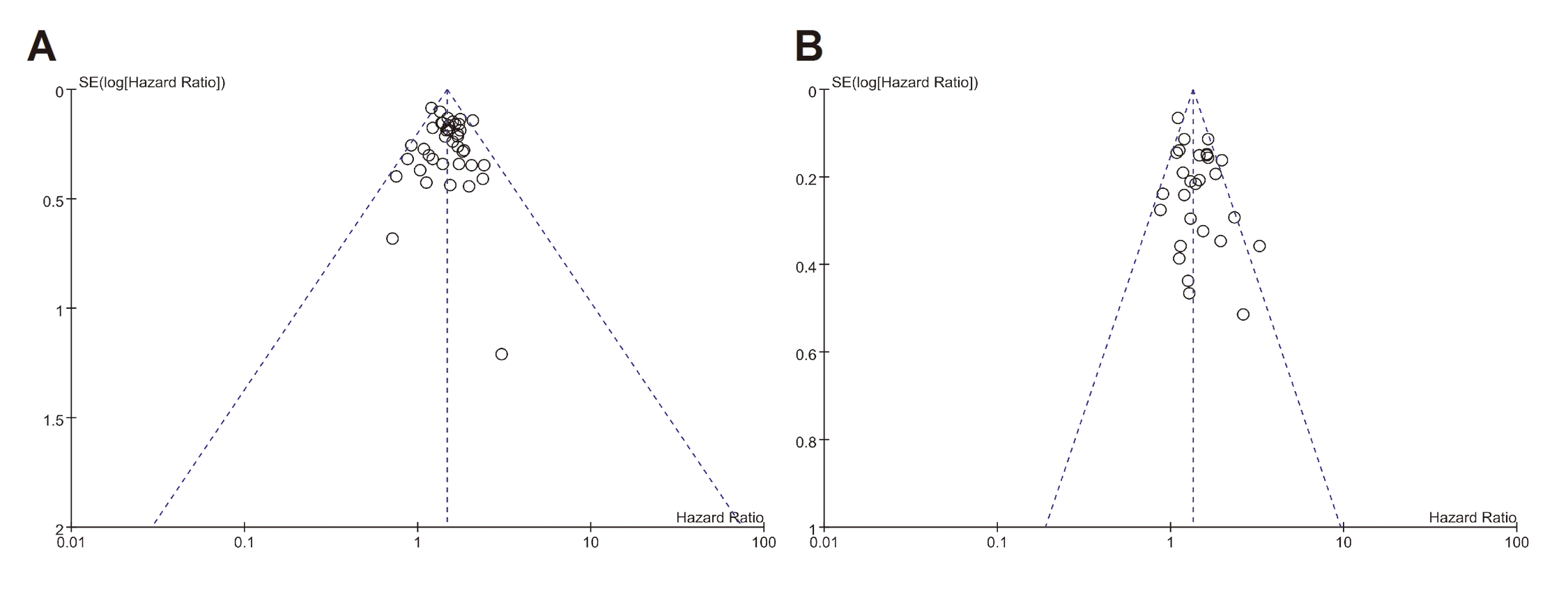

Supplement: S4 Fig — (TIF) [file pone.0180513.s007.tif]
